# Feasibility of a customizable training environment for neurointerventional skills assessment

**DOI:** 10.1371/journal.pone.0238952

**Published:** 2020-09-17

**Authors:** Marie Teresa Nawka, Uta Hanning, Helena Guerreiro, Fabian Flottmann, Noel Van Horn, Jan-Hendrik Buhk, Jens Fiehler, Andreas Maximilian Frölich

**Affiliations:** Department of Diagnostic and Interventional Neuroradiology, University Medical Center Hamburg-Eppendorf, Hamburg, Germany; National University of Ireland - Galway, IRELAND

## Abstract

**Objective:**

To meet increasing demands to train neuroendovascular techniques, we developed a dedicated simulator applying individualized three-dimensional intracranial aneurysm models (‘HANNES’; Hamburg Anatomic Neurointerventional Endovascular Simulator). We hypothesized that HANNES provides a realistic and reproducible training environment to practice coil embolization and to exemplify disparities between neurointerventionalists, thus objectively benchmarking operators at different levels of experience.

**Methods:**

Six physicians with different degrees of neurointerventional procedural experience were recruited into a standardized training protocol comprising catheterization of two internal carotid artery (ICA) aneurysms and one basilar tip aneurysm, followed by introduction of one framing coil into each aneurysm and finally complete coil embolization of one determined ICA aneurysm. The level of difficulty increased with every aneurysm. Fluoroscopy was recorded and assessed for procedural characteristics and adverse events.

**Results:**

Physicians were divided into inexperienced and experienced operators, depending on their experience with microcatheter handling. Mean overall catheterization times increased with difficulty of the aneurysm model. Inexperienced operators showed longer catheterization times (median; IQR: 47; 30-84s) than experienced operators (21; 13-58s, p = 0.011) and became significantly faster during the course of the attempts (rho = -0.493, p = 0.009) than the experienced physicians (rho = -0.318, p = 0.106). Number of dangerous maneuvers throughout all attempts was significantly higher for inexperienced operators (median; IQR: 1.0; 0.0–1.5) as compared to experienced operators (0.0; 0.0–1.0, p = 0.014).

**Conclusion:**

HANNES represents a modular neurointerventional training environment for practicing aneurysm coil embolization in vitro. Objective procedural metrics correlate with operator experience, suggesting that the system could be useful for assessing operator proficiency.

## Introduction

Over the last decades, endovascular therapy of intracranial aneurysms (IA) using coil embolization has been established as a standard treatment approach [1]. Owing to the wide range of materials and devices available, a well-founded education for beginning neurointerventionalists and constant practice for advanced physicians is indispensable, aiming to maintain high neurointerventional standards [2, 3]. Simulation technology for medical teaching and training can provide opportunities to learn and enhance medical skills [2]. There is a growing array of neurointerventional setups to teach and train distinct endovascular procedures, either operating with silicone models or by means of rapid prototyping technologies [4–6]. Among the available training models and simulators, anatomically precise three-dimensional (3D) printed IA models integrated into in vitro training environments [7, 8] represent a unique way of translating actual clinical challenges into a protected training environment. Trainees can practice with catheters and devices used in clinical routine and receive direct haptic feedback. In vitro training can thus potentially help to increase patient safety [7].

Any form of structured training requires a system to assess trainees’ progress. The goal of the present analysis was to assess the capabilities of a recently developed modular, customizable in vitro training environment utilizing 3D printed IA models (‘HANNES’; Hamburg Anatomic Neurointerventional Endovascular Simulator) [9] for discerning trainees of different skill levels. We aimed to identify procedural characteristics that may serve as markers of operator proficiency.

The overall goal of this endeavor is the development of a system for objective performance comparison between operators, which could be an integral part of neurointerventional educational curricula in practice.

## Methods

Research was approved by the local ethical committee at Medical Chamber Hamburg, Germany. Individual consent was waived because the data were analyzed anonymously.

Two radiology residents (first year), two board-certified radiologists in neuroradiology fellowship training and two board-certified radiologists with subspecialty certification in neuroradiology were recruited into a standardized training procedure. One of the board-certified radiologists in neuroradiology fellowship and both board-certified neuroradiologists had clinically performed coil embolization.

For statistical analysis, participants were divided into two groups depending on their clinical experience with handling of intracranial microcatheters: inexperienced (no prior experience with the handling of intracranial microcatheters) and experienced (familiar in handling intracranial microcatheters as first operator).

The training procedure consisted of catheterization of three IA (two internal carotid artery (ICA) aneurysms and one basilar tip aneurysm) and placing a framing coil into the aneurysm sac of each model. Finally, one predetermined ICA aneurysm model had to be fully embolized using coils. The experiments were conducted separately in a research laboratory with HANNES. Participants received no external guidance.

### Endovascular simulation with HANNES

**Fig 1:**
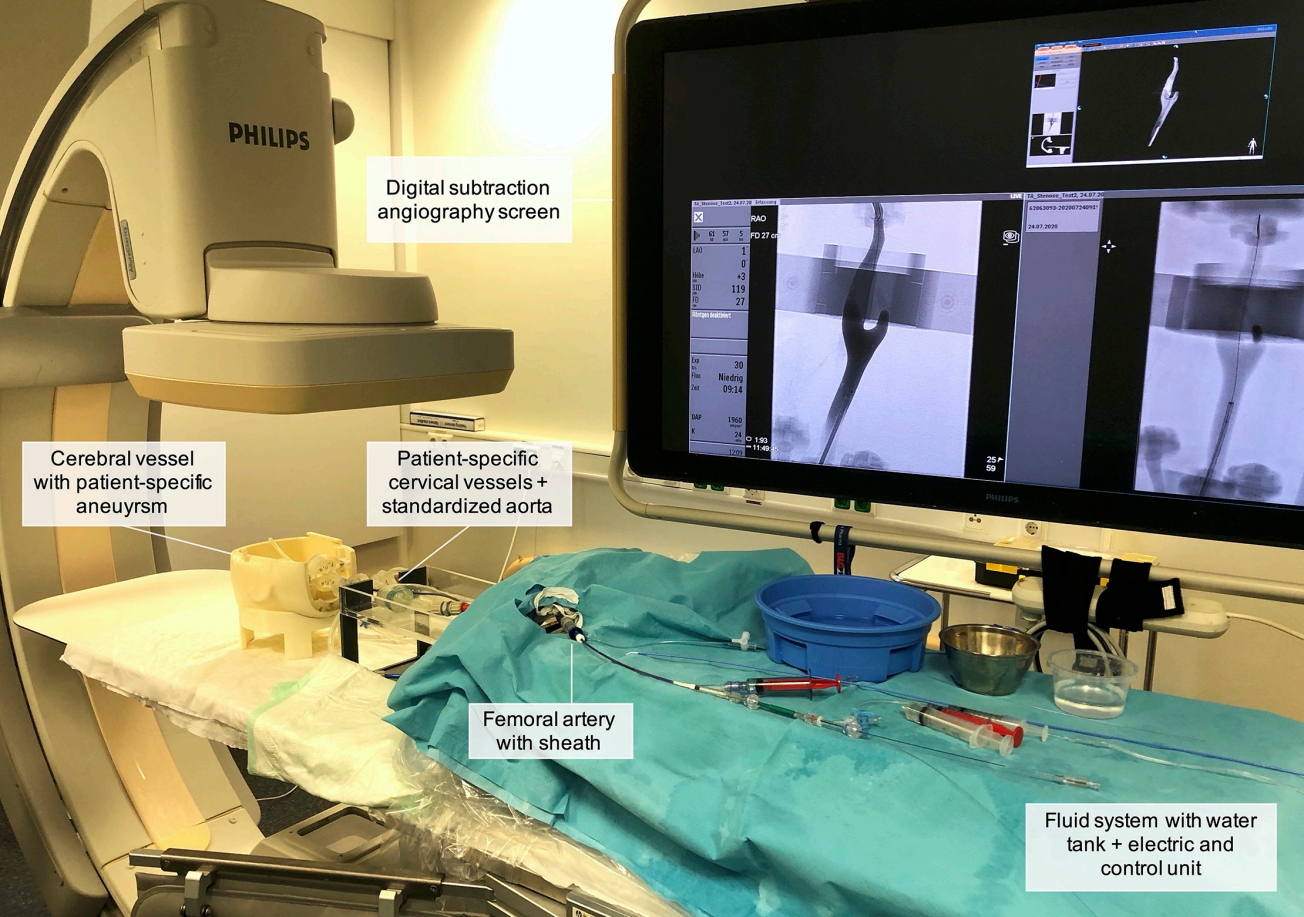
Image of HANNES in the research laboratory. The simulator is fabricated of different units including the electric and control element, the fluid system including a water tank, the standardized aorta and the patient-specific cervical and intracranial vessels.

Technical features of the neurovascular training environment HANNES have been described previously [9]. In short, a modular vascular phantom comprising the complete access route from the femoral artery to the circle of Willis was perfused with heated water at a temperature of 37°C. To reduce surface friction, commercial soap was added into the system. The configuration provides a flow rate of around 0.4L/min through the intracranial vessels, and a pulse rate of 70 bpm [9]. The thoracoabdominal vasculature was replicated by a commercially available silicone model (United Biologics, Inc., California, USA). Cervical vasculature and three intracranial aneurysms were attained as quickly interchangeable 3D printed models fabricated by the investigators. Fig 1 illustrates HANNES and gives detailed information on the different parts of the simulator.

Trials were conducted using a dedicated experimental angiography system (Allura Clarity FD 20, Philips Healthcare, Best, The Netherlands). Fluoroscopy was continuously recorded for each experiment.

### Aneurysm models

Three additively manufactured intracranial aneurysm models were implemented according to fabrication standards described previously [9]. Based on 3D rotational angiography data, models were constructed digitally and fabricated with laser stereolithography using an opaque material with a wall thicknesses of 1 mm (formlabs flexible photopolymer resin FLFLGR02 and form2 printer, Formlabs Inc., Somerville, MA, USA) [9]. Models of three saccular aneurysms were obtained: #1: basilar tip, 9.0x8.0x8.0mm, 4.5mm neck width; #2: ICA “carotid-T”, 5.2x3.9x4.8mm, 2.8mm neck width; #3: ICA paraophthalmic, 7.6x6.4x5.9mm, 3.9mm neck width.

### Procedures

Prior to the experiments, all participants were instructed in the basics of the coiling procedure, including explanation of the radiopaque coil markers, accurate coil positioning and coil detachment. All operators were familiar with the angiography system, flushing systems and y-adapters. Participants were blinded to the evaluation criteria.

All experiments were performed on HANNES. The setup was prepared before each attempt, aneurysm model exchange was done during the experiments. Through an 8F right femoral artery sheath (Cordis Corporation, Miami, Florida, USA), a 7F guiding catheter (Vista Brite Tip, Cordis Corporation, Miami, Florida, USA) was pre-positioned in the distal cervical internal carotid artery or distal V2 segment of the vertebral artery, respectively. 3D rotational angiography was obtained. A Headway 17 microcatheter (MicroVention, Tustin, California, USA) and Transend EX microwire (Boston scientific, Marlborough, Massachusetts, USA) were then advanced to the guide catheter tip; this setup represented the starting position for all experiments. Prior to the experiments, each operator measured the aneurysm on the rotational angiogram and chose a working projection. All participants catheterized the aneurysms in the same order from #1 to #3. In each model, participants were given the following tasks: 1) Select the aneurysm with the microcatheter and microwire to obtain an intrasaccular position adequate for placing the first coil. Repeat this three times from the starting position. 2) After the third selection attempt, choose and introduce an appropriate first coil, trying to obtain an adequate framing position. 3) Only in model #3: Detach the framing coil and introduce and detach further coils to embolize the aneurysm. Only 3-dimensional coils (sizes between 2–12 mm) of uniform standard stiffness were available (Axium Coils, Medtronic, Minneapolis, Minnesota, USA). A maximum time of 600s (10 minutes) was allowed for each experiment. In case of exceeding the time limit, the task was labelled as ‘failed’.

### Fluoroscopy video analysis

For each operator and each aneurysm model, mean probing times were taken, starting from the initial microcatheter position as described above until a satisfying intrasaccular position for placement of the first coil was achieved. The end position was verbally confirmed by the operator, catheterization time was recorded in seconds (s). Additionally, times for framing coil placement and mean times of the complete coiling process for aneurysm model #3 were collected. Occurrence of the following potentially dangerous maneuvers were analyzed by the most experienced board-certified radiologist with subspecialty certification in neuroradiology (8 years of experience): 1) Erratic wire or microcatheter movements against the aneurysm wall, 2) microwire or microcatheter side branch access into the ophthalmic, anterior choroidal or posterior communicating artery ostia, 3) instrument movement without using fluoroscopy, 4) leading with a short segment of wire at the distal end of the microcatheter, 5) inversion of the microcatheter, 6) aneurysm rupture, dislocation of the microcatheter from the aneurysm sac and permanent dislocation of coils into the parent vessel. The position of the framing coil was visually graded: optimal (1), if an ideal framing could be achieved. Acceptable (2), if there was one or fewer coil loops across the aneurysm neck. Insufficient (3) in cases of coil prolapse into the parent vessel or absent aneurysm neck coverage. Occlusion status of the coiled aneurysm model #3 was assessed after the procedure, applying the modified Raymond-Roy Classification (MRRC) considering the following possible results: Class I = complete obliteration, class II = residual neck, class III = residual aneurysm [10]. As some remaining contrast opacification within the coil mesh could uniformly be observed in our in vitro environment, classes IIIa and IIIb were excluded [10].

### Statistics

Numerical results are labeled as mean±SD. To test for normal distribution of the data, we performed the Shapiro-Wilk test. As the data did not follow normal distribution, group comparison for the variables ‘catheterization time’, ‘dangerous maneuvers’, ‘time of framing coil placement’ and ‘results of framing coil’ was performed using the Mann-Whitney U test. To assess the relationship between the operator’s experience and the catheterization times, rank correlation was performed, selecting Spearman’s rho as a correlation coefficient; rho was interpreted according to Chan et al [11]. Analyses were performed using MedCalc Statistical Software (University of South Carolina, Columbia, South Carolina, USA) and MS Excel 2016 (Microsoft, Redmond, USA). A p-value of <0.05 was determined as statistically significant, significant results in Table 1 are marked with an asterisk (*).

**Table 1:** Comparison of the variables ‘catheterization time’, ‘dangerous maneuvers’, ‘time of framing coil placement’ and ‘framing coil results’, stratified by inexperienced and experienced operators.

Variable	Group 1 = inexperienced operators	Group 2 = experienced operators	Group comparison
			p-value
**Total catheterization time (s), median; IQR**			
Model #1-#3	47; 30–84	21; 13–58	p = 0.011*
**Dangerous maneuvers (n), median; IQR**			
Model #1-#3	1.0; 0.0–1.5	0.0; 0.0–1.0	p = 0.014*
**Framing coil placement (s), median; IQR**			
Model #1-#3	76; 41–91	49; 30–76	p = 0.341
Result of framing coil**, median; IQR			
Model #1-#3	2; 1–3	1; 1–1	p = 0.018*
**Result of complete coiling (Raymond-Roy, model #3 only)**			
Operators 1, 2, 3	III, II, III	II, II, II	NA

**1 = optimal framing, 2 = acceptable framing, 3 = insufficient framing.

*significant values in the group comparison.

NA = not applicable.

## Results

Two first-year radiology residents and one board-certified radiologist in neuroradiology fellowship were grouped as ‘inexperienced’ operators (zero years of neurointerventional experience). One board-certified radiologist in neuroradiology fellowship and the two board-certified neuroradiologists who were familiar with the use of microcatheters were grouped as ‘experienced’ operators (mean±SD: 4±3 years of experience).

Table 1 provides an overview of all endpoints assessed, stratified by inexperienced (group 1) and experienced (group 2) physicians. Two inexperienced operators failed to place a framing coil into aneurysm models #2 and #3. Framing coil sizes and filling coils for the complete coiling process of model #3 are reported in the S1 Table.

### Procedural time metrics

Mean overall catheterization times were 37±24s for aneurysm model #1, 44±31s for aneurysm model #2 and 81±74s for aneurysm model #3. Inexperienced operators showed significantly longer probing times for all three aneurysm models (median; IQR: 47; 30-84s) than experienced operators (21; 13-58s, p = 0.011). In our analysis, the inexperienced physicians became significantly faster during the course of the attempts (rho = -0.493, p = 0.009), whereas the experienced operators showed only a trend towards improvement of catheterization times over time (rho = -0.318, p = 0.106). We observed a poor to moderate negative correlation between the duration of catheterization and the operator’s experience (rho = -0.306, p = 0.010).

**Fig 2:**
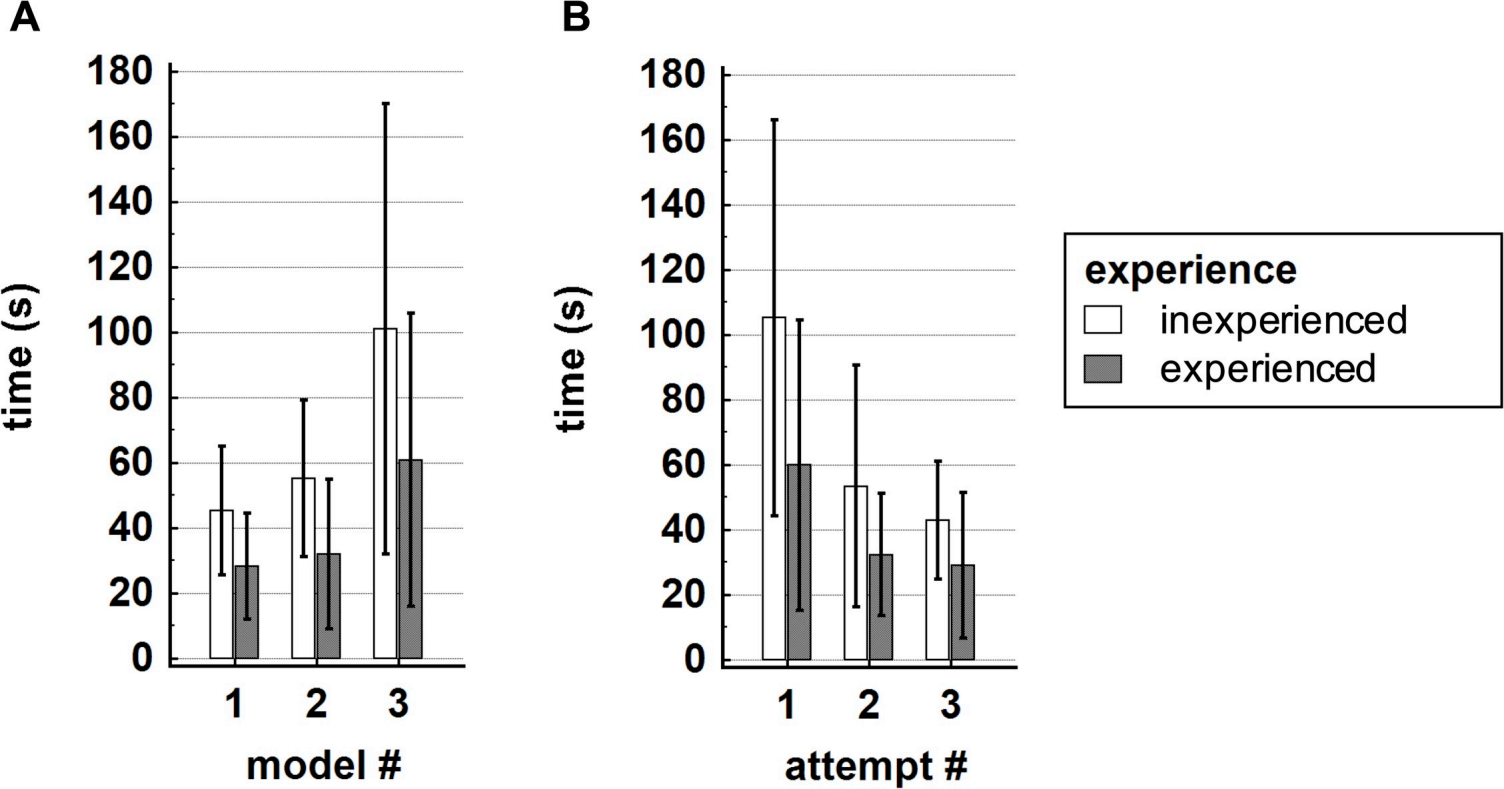
Bar charts illustrate catheterization times, operator experience, aneurysm models and attempts. (A) Experienced operators showed a clear trend towards shorter catheterization times than inexperienced physicians. Both groups needed longer to catheterize the most challenging aneurysm model #3. (B) Mean catheterization times per attempt for all aneurysm models, grouped by the operator’s experience. Faster catheterization times were observed for both inexperienced and experienced operators with consecutive attempts, but this correlation was significant for the inexperienced physicians only.

Fig 2 illustrates the relationship between time metrics, models, attempts and operator experience.

### Dangerous maneuvers

An eccentric position of the microcatheter during the coiling procedure in aneurysm model #3 was observed in 4 operators. Coil prolapse into the parent vessel as a final result after complete coil embolization of model #3 occurred in two cases. Inadvertent side branch access was observed several times in five operators in model #3 and once in model #2. One of the inexperienced physicians lead the catheter with a short segment of wire throughout catheterization of all models. No microcatheter inversions, material manipulation without fluoroscopic guidance or aneurysm ruptures were observed. The inexperienced operators showed significantly higher numbers of dangerous maneuvers throughout all attempts (median; IQR: 1.0; 0.0–1.5) compared to the experienced operators (median; IQR: 0.0; 0.0–1.0, p = 0.014).

### Coiling results

**Fig 3:**
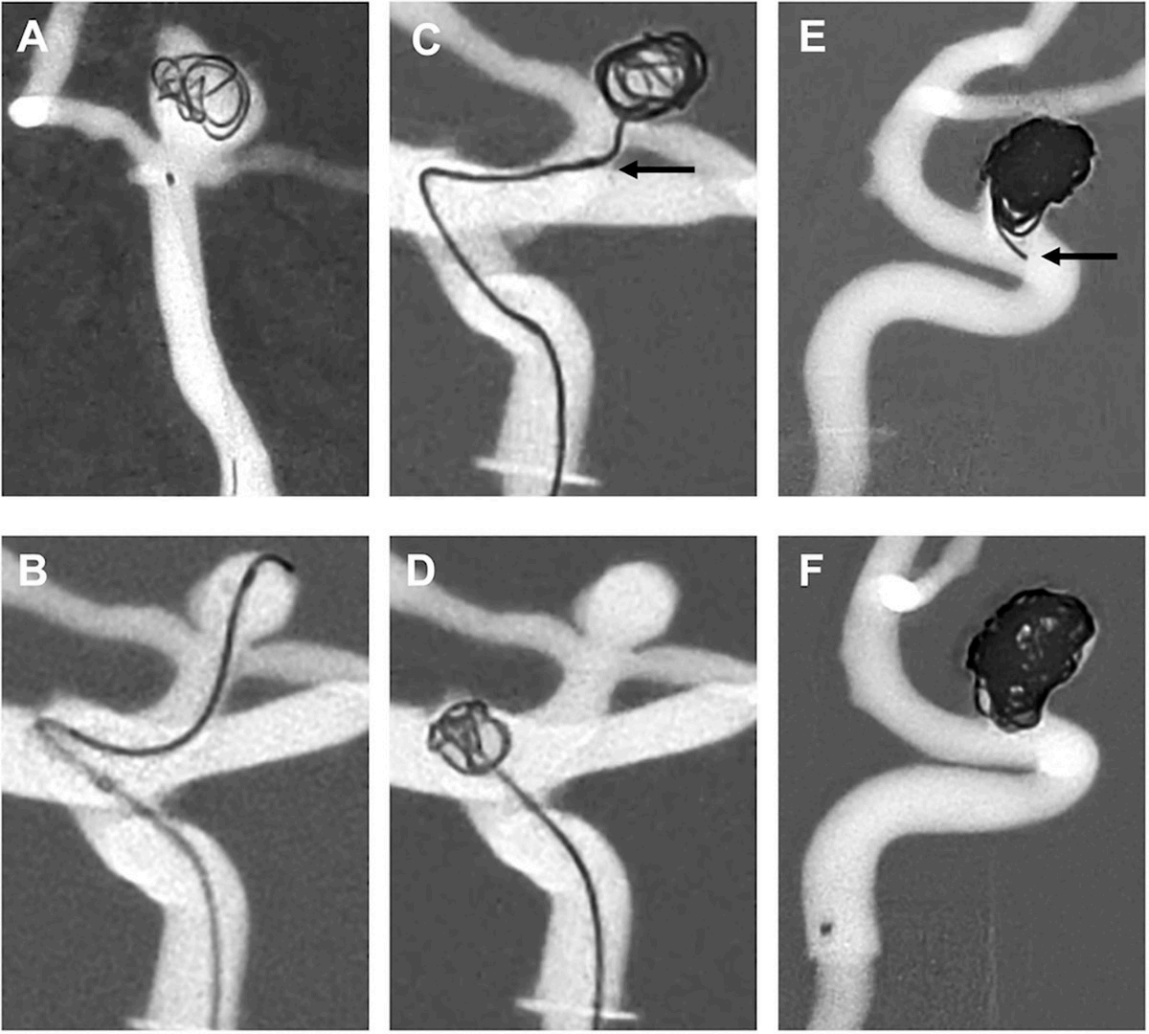
Spectrum of results and complications during coil embolization. (A) Final framing coil position in aneurysm model #1, coil placement performed by an inexperienced operator, result graded as insufficient (3). (B) Erratic wire movement against the aneurysm wall of model #2 during aneurysm catheterization by one inexperienced operator. (C) Dislocation of the microcatheter from the aneurysm sac during placement of the framing coil, performed by an inexperienced operator. (D) Dislocation of the framing coil into the parent vessel during coil placement, performed by one inexperienced operator. (E) Coiling result of one inexperienced operator, showing a residual neck (Raymond-Roy Occlusion Classification II), arrow indicating coil prolapse into the ICA. (F) Coiling result of the most experienced operator, showing a residual neck (Raymond-Roy Occlusion Classification II).

Two inexperienced operators failed to place a framing coil into aneurysm models #2 and #3. Hence, one inexperienced operator failed at completing coil embolization of model #3. Choice of framing coils differed between the physicians, ranging from 3mm x 8cm– 8mm x 30cm for model #1, 4mm x 12cm– 6mm x 20cm for model #2 and 6mm x 20cm– 7mm x 30cm for model #3. Inexperienced operators showed significantly poorer framing coil results (2; 1–3) compared to the experienced operators (1; 1–1, p = 0.018). Two of the inexperienced operators showed a residual aneurysm (III) and one showed a residual neck (II), whereas all three experienced operators generated residual necks after coiling (III). In two operators (one inexperienced and one experienced operator) coil prolapse into the parent vessel occurred at the end of the procedure. Fig 3 displays a range of results and complications which could be overserved throughout the experiments in aneurysm models #1 to #3.

## Discussion

In the present study, we have utilized a modular, customizable endovascular in vitro training environment to assess the performance of a group of physicians during basic steps of aneurysm coil embolization. Our results indicate that the presented setup is suitable for performing a relatively large number of such focused experiments within an acceptable time frame and generate objective data that might serve as surrogate markers of operator performance. We observed differences between inexperienced and experienced operators for several procedural metrics, including the overall catheterization time, occurrence of unwarranted or dangerous maneuvers as well as increasing speed in aneurysm selection with growing operator experience. A variety of complications could be observed, including microcatheter displacement from the aneurysm sac, coil prolapse into the parent vessel and failure to position coils. Thus, it seems possible to utilize the presented framework not only to educate and train physicians in interventional technique, but also to assess individual operators’ proficiency.

The beneficial role of educational simulation in various medical fields has been previously demonstrated [2, 3, 6, 7]. Amid the ongoing development of neuroendovascular devices for the treatment of IA, suitable training opportunities for neurointerventionalists are indispensable. In vitro training enables physicians to practice techniques in safe surroundings and intimately familiarize themselves with the behavior of endovascular devices and may thus contribute to patient safety [12]. The role of in vitro training as a part of physician education in interventional neuroradiology can certainly be further expanded. The idea of using silicone aneurysm models in a laboratory set-up to train intracranial coil embolization has been conceived earlier [13], and our results support this concept. Given that inexperienced physicians showed a greater increase in aneurysm selection speed than experienced operators and were also more likely to arrive at complications such as catheter dislocation, it seems that a part of the learning curve required for microcatheter navigation may be practiced in this training environment.

The current experiments were not designed as a curriculum to teach the basics of coil embolization. However, utilizing the present framework it certainly seems feasible to develop structured training scenarios, covering the fundamentals of coil embolization and other endovascular techniques. Cases could be selected to represent the spectrum of clinically encountered aneurysm morphologies and locations. Ideally, such curricula would be accompanied by a theoretical background focusing on technical procedural aspects that can be reproduced in the in vitro training environment. These encompass the safe handling of catheters, y-adapters, flushes and wires, adequate selection and navigation of catheters along the vascular tree and also include the fundamentals of selecting, sizing and delivering different neurovascular implants. Objective evaluation of procedural metrics similar to what is demonstrated here may then supplement training programs, ensuing that training goals are met. Video analysis for detailed evaluation of each procedure can be a valuable tool, in this regard.

As intraoperative complications such as aneurysm perforation or thromboembolism can have fatal consequences, safe handling of neurointerventional materials is crucial [14]. Hence, not only optimal preoperative preparation of the patient–e.g. adequate indication, antiplatelet therapy etc.–but moreover a well-trained physician with sufficient technical skills are pivotal factors for procedural success [14]. The improvement of technical skills by means of simulator based angiography in neurosurgery has been previously demonstrated by Fargen et al. [15]. They describe a training course for inexperienced residents, focusing on simulator practice for learning basic endovascular skills [15]. Objective assessments showed a decrease in time needed to perform a four vessel angiogram [15]. As opposed to our study comparing two groups of different expertise, they assessed the learning curve of inexperienced physicians who were given detailed lectures and instructions in advance and utilized a computer-based simulator as opposed to our in vitro approach [15]. Computer simulation was also employed in a study by Spiotta et al., who evaluated the feasibility of endovascular simulator training within a neurosurgical residency program [3]. Consistent with our data, they observed inferior performance of inexperienced physicians, resulting in a higher number of potentially dangerous maneuvers compared to experienced physicians [3]. In contrast to computer simulators, in vitro training currently requires fluoroscopic control, limiting flexibility. Catheters and devices cannot always be reused which may increase costs, although the total costs of training depend on a multitude of factors including frequency of use and type of training. On the other hand, in vitro training offers the key benefit of using actual clinical treatment instruments, therefore providing a much more realistic haptic experience. This advantage is particularly relevant for newer endovascular devices such as flow diverters and flow disruptors, where an intimate knowledge of the forces associated with device deployment is required in order to avoid technical complications such as inadequate positioning [16]. It is our belief that the advantage of realistic haptic feedback obtainable with in vitro training is well worth the effort. However, to our knowledge, there are no data directly comparing the training effects of computer simulators and in vitro training yet.

A possible further direction of research is the refinement of in vitro environments to broaden their applicability. For instance, models might be designed that are compatible with both endovascular and microsurgical techniques, increasing their relevance particularly for physicians learning both techniques [3, 15, 17, 18]. Beyond aneurysm treatment, HANNES might also be suitable for training mechanical thrombectomy [19]. Different in vitro settings to practice mechanical thrombectomy in stroke have been developed [20–23], but their benefit needs to be further assessed. Moreover, HANNES could be valuable to objectively assess supervisors’ abilities in the course of training thrombectomy by remote live streaming support, as this concept has been investigated to support less experienced neurointerventionalists earlier [24].

Limitations include the small number of aneurysms in this pilot study, all with saccular morphology and absence of certain challenging features such as partial thrombosis. The number of operators in each group is small. Inclusion of more operators with different expertise levels might better enlighten to degree differences in performance can be objectively be observed. Current in vitro training is furthermore limited by the absence of real blood and reactive vasculature. Aneurysm rupture was highly unlikely in the models we produced, although it would be possible to fabricate models with thinner, more fragile walls. Vasospasm, dissection and thrombosis will not occur and are very important complications to master clinically. However, these shortcomings do not speak against performing in vitro training, as a supplement to clinical training. To shorten the overall duration of experiments, it was decided to focus on intracranial aneurysm selection and coil placement. Inclusion of catheter preparation, percutaneous access and guide catheter placement as part of the experimental tasks might have yielded different results.

## Conclusion

This analysis demonstrates the feasibility of a modular neurointerventional training environment for practicing aneurysm coil embolization in vitro. Objective procedural metrics correlate with operator experience, suggesting that the system could be useful for assessing operator proficiency.

## Supporting information

S1 Table Framing coil sizes and complete coiling results for aneurysm models #1-#3, stratified by inexperienced and experienced operators. (PDF)
