# Supplementary material for: Feasibility of a customizable training environment for neurointerventional skills assessment
Source: PLoS One. 2020 Sep 17;15(9):e0238952. doi: 10.1371/journal.pone.0238952 (PMC7498089; doi:10.1371/journal.pone.0238952)
Supplement: S1 Table — (PDF) [file pone.0238952.s001.pdf]

S1 Table. Framing coil sizes and complete coiling results for aneurysm models #1-#3, stratified by inexperienced and experienced operators. In models #1 and #2 only a framing coil was placed, aneurysm model #3 was coiled completely.

Aneurysm model	Framing coil, size – group 1 = inexperienced operators	Framing coil, size – group 2 = experienced operators
**Model #1 (framing coil only)**		
Operator 1	3mm x 8cm	8mm x 30cm
Operator 2	8mm x 30cm	8mm x 30cm
Operator 3	9mm x 20cm	8mm x 30cm
**Model #2 (framing coil only)**		
Operator 1	6mm x 20cm (failed to place framing coil)	4mm x 12cm
Operator 2	5mm x 15cm	5mm x 15cm
Operator 3	6mm x 20cm	6mm x 20cm
**Model #3 (framing coil* and complete coiling process)**		
Operator 1	6mm x 20mm*, 4mm x 12cm, 4mm x 12cm	7mm x 20cm*, 5mm x 15cm, 3mm x 8cm, 2mm x 6cm (x2)
Operator 2	7mm x 30cm*, 5mm x 15cm, 2mm x 6cm	7mm x 30cm*, 5mm x 15cm, 4mm x 12cm
Operator 3	7mm x 20cm*, failed to place faming coil and complete coiling process	7mm x 30cm*, 4mm x 12cm, 3mm x 8cm, 2mm x 6cm
